# Effects of aerobic exercise and resistance training on lipid profiles and inflammation status in patients on maintenance hemodialysis

**DOI:** 10.4103/0971-4065.73442

**Published:** 2010-10

**Authors:** R. Afshar, L. Shegarfy, N. Shavandi, S. Sanavi

**Affiliations:** Department of Nephrology, Faculty of Medicine, Shahed University, Mustafa Khomeini Hospital, Iran; 1 Department of Sport Physiology, Arak University, Iran; 2 Clinical Fellow of Nephrology, Internist, University of Social Welfare and Rehabilitation Sciences, Akhavan Center, Iran

**Keywords:** Aerobic exercise, resistance training, physical function, hemodialysis

## Abstract

Physical function limitation is a common disorder in chronic hemodialysis (HD) patients, relating to increased morbidity and mortality. The aim of this study was to determine the effects of aerobic and resistance trainings on blood lipids and inflammation status in HD patients. Out of 30 volunteer males who had been undergoing conventional maintenance HD within an HD unit in Tehran, 21 subjects were enrolled. They were randomly assigned into aerobic exercise group – resistance training group undergoing an 8-week intradialytic exercise program (three times/week) and control group (*n* = 7, each). Training program consisted of 10-30 min stationary cycling at an intensity of 12–16 out of 20 at the rate of perceived exertion (RPE) of Borg scale in aerobic group and using ankle weights for knee extension, hip abduction and flexions at an intensity of 15–17 out of 20 at the RPE of Borg scale in resistance group. Fasting blood samples for serum biochemistry were drawn at baseline and 8 weeks. The age, HD duration, and physical activity score were 51.6±18.9yrs; 25.1±13.9 mo, and 19.2±7.6, respectively. Diabetes mellitus (43%), hypertension (28%), and obstructive uropathy (14%) were the most common underlying diseases. Aerobic and resistance exercises were correlated with serum creatinine (*P*< 0.0001 and *P*<0.001) and hs-CRP levels (*P*=0.005 and *P*=0.036) reduction so that aerobic exercise induced more reduction. These exercises had no influence on weight, Kt/V values, serum urea, albumin, hemoglobin, and lipid levels (*P*>0.05). Both intradialytic aerobic and resistance exercises showed beneficial effects on inflammation status without any influences on serum lipid levels probably due to short duration of the study which was not accompanied with body weight changes. Solute removal had no change during exercise programs. There is a need for more investigation on the role of exercise in HD patients.

## Introduction

End-stage renal disease (ESRD) patients have limited physical functioning as assessed by subjective reporting,[1] peak oxygen consumption,[2–5] and physical performance and muscle strength tests.[6,7] About one-third of hemodialysis (HD) patients are unable to perform the normal daily activities without assistance.[8] On the other hand, physical functioning has been shown to be a major determinant of the quality of life.[9–11] Thus, interventions to improve functioning in this population have the potential to improve quality of life.

In ESRD patients, exercise has beneficial effects on functional capacity, anemia, cardiovascular risks factors, dyslipidemia, and psychosocial problems.[12] However, few patients are able or willing to participate in an exercise training program organized on an outpatient basis.[9,12,13] Several studies have been performed regarding the effects of various exercises in HD patients, particularly on the nondialysis days.[14] It has been suggested that exercise could improve solute removal during dialysis by increasing muscle blood flow, which results in greater efflux of uremic toxins into the vascular compartment.[7,15]

This randomized controlled study was designed to determine the effects of intradialytic aerobic exercise and resistance (strength) training on lipid profiles and inflammation status in HD patients.

## Methods

The study population was composed of 21 males (because of religious beliefs and location limitation for gender separation during exercise training, the women refused to participate) undergoing conventional maintenance HD (three times per week), who were between the ages 28–74 years, in an HD unit in Tehran. Among 60 HD patients, 30 subjects volunteered to participate in this study and with regard to their medical history, 21 patients were enrolled. Informed consent was obtained from all participants and they were randomized to resistance training (*n*=7), moderate intensity aerobic training (*n*=7), and control groups (*n*=7).

The inclusion criteria were as follows: maintenance HD > 3 months; age > 20 years; good compliance with the dialysis treatment (not missing more than two dialysis sessions in the prior month); and absence of lower extremity dialysis graft. The exclusion criteria were presence of active infection or inflammation, autoimmunity disorders, and malignancy; presence of severe muscle weakness or interfering skeletal deformity; history of repeated episodes of hypoglycemia; cardiopulmonary contraindications to resistance exercise such as myocardial infarction within prior 6 months, active angina, and uncompensated congestive heart failure; hospitalization during prior month; cerebrovascular accidents within prior 6 months; and history of prior regular exercise training.

The training program consisted of a 5-min warm up, a 10–30-min aerobic or resistance training, and a 5-min cool down period during the first 2 h of each dialysis session in recumbent position, within 8 weeks. According to primary results of Baecke questionnaire on physical activity which was filled for all participants at baseline, aerobic training participants should perform stationary cycling at an intensity of 12–16 out of 20 at the rate of perceived exertion (RPE) of Borg scale so that intensity involved 65–85% of an individual’s maximal capacity, a level at which cardiovascular health can be obtained. The Borg scale is a simple method of RPE and can be used by coaches to gauge an athlete’s level of intensity in training and competition. There are a number of RPE scales but the most common are the 15-point scale (6–20) and the 9-point scale (1–10).[16]

Resistance exercise training of the lower extremities was performed in three sets and under the supervision of a physician by applying ankle weights for knee extension-flexion and hip abduction-flexion at an intensity of 15–17 out of 20 at the RPE scale. Starting weights were determined from a three-repetition maximum (3RM) using ankle weights that can be adjusted in 0.5–1 kg/week increments. A 3RM is the maximum weight that can be lifted three times with a proper technique. Training started at approximately 60% of 3RM for two sets of eight repetitions and was increased to three sets as tolerated. When patients could perform three sets successfully, the weight was increased. Blood pressure and heart rate of the participants were monitored each 5 min during exercise. Fasting venous blood samples were obtained from patients before mid-week dialysis session in order to measure serum urea, creatinine, albumin, hemoglobin, lipid levels [low density lipoprotein (LDL) cholesterol, high-density lipoprotein (HDL) cholesterol, and triglyceride], and CRP (turbidometric technique with normal range below 10 mg/L), at baseline and 8 weeks.

Urea clearance was calculated by logarithmic single pool *Kt/V* (*spKt/V*) equation (at baseline and 8 weeks) according to the Daugirdas formula:[17]

$$\mathrm{spKt}/V\;=\;\left(-l\;n\;\left[R-0.008\times t\right]\;+\;4-3.5\times R\right)\;\times \;\mathrm{UF}/W$$

Where *R* is the post–pre SUN (serum urea nitrogen) ratio, *t* is session length (in h), *UF* is the volume of fluid removed during dialysis (in liters), and *W* is postdialysis body weight (in kg). Pre- and postdialysis (immediately at the end of dialysis) blood samples were drawn to obtain respective serum urea concentrations, in order to calculate *spKt/V*. The minimum target dose of *spKt/V*, which is recommended by Kidney Disease Quality Outcomes Initiative (K/DOQI) is 1.2.[16] In order to calculate *spKt/V*, the postdialysis blood sample was drawn from arterial blood line 20 sec after dialysis session termination while the pump speed was reduced to 80 mL/min.

Data analyses were performed with the SPSS, version 16 (SPSS Inc., Chicago, IL, USA) and *t-test*, ANOVA test, and Pearson’s correlation test were used. Significance was accepted at *P*<0.05.

## Results

The most common causes of ESRD groups were diabetes mellitus (43%), hypertension (28%), unknown etiology (15%), and obstructive uropathy (14%), respectively. Participants’ demographic characteristics in three study groups have been shown in Table 1.

**Table 1:** Participants’ characteristics in different groups

Parameters	Aerobic training group	Resistance training group	Control group
Age (years)	50.7±21.06	51±16.4	53±19.4
Body mass index (kg/m^2^)	22.71±2.98	21.96±1.41	22.3±2.18
Hemodialysis duration (months)	25.71±7.61	24.86±18.69	24.86±15.44
Physical activity (Baecke score)	20.83±9.17	18.14±6.78	18.82±6.91

Values are Mean ± SD

One-way ANOVA test revealed that BMI did not significantly change in three groups during 8 weeks. Compared to control group, a significant reduction of serum creatinine (*P*<0.0001 and *P*<0.001) and hs-CRP (*P* =0.005 and *P* =0.036) was demonstrated in aerobic and resistance training groups. There was statistically significant difference between aerobic exercise and resistance training in above effects (*P*<0.001, each). Furthermore, these exercises had no influence on serum urea, albumin, hemoglobin, and lipid levels including LDL-C, HDL-C, and triglyceride (*P*>0.05). Table 2 shows serum biochemistry and *Kt/V* values at baseline and 8 weeks.

**Table 2:** Serum chemistry values in three groups at baseline and end of study

Serum parameters	Groups	Baseline	End of study
Creatinine (mg/dL)	Control	9.11±3.25	9.22±3.12
	Aerobic exercise	11.1±2.49	3.82±1.67
	Resistance training	10.643±2.38	4.27±2.54
spKt/V	Control	1.09±0.26	1.1±0.25
	Aerobic exercise	1±0.31	1±0.33
	Resistance training	1.11±0.26	1.12±0.3
Urea (mg/dL)	Control	101±12	101±12.2
	Aerobic exercise	97.7±13.3	90.7±10.8
	Resistance training	99.8±12.8	91.8±11.5
Hemoglobin (g/dL)	Control	10.3±0.3	10.2±0.3
	Aerobic exercise	10.1±0.5	10.1±0.5
	Resistance training	10.5±0.2	10.3±0.2
Albumin (g/dL)	Control	4±0.3	4±0.3
	Aerobic exercise	4±0.4	4±0.4
	Resistance training	4±0.3	4±0.3
hs-CRP (mg/L)	Control	4.08±3.98	4.14±3.87
	Aerobic exercise	5.45±2.49	0.88±0.59
	Resistance training	7.07±2.87	2.27±1.79
Triglyceride (mg/dL)	Control	224±100.65	225.71±79.97
	Aerobic exercise	209±56.93	185.14±61.78
	Resistance training	188.57±96.33	145.71±61.86
HDL-C (mg/dL)	Control	32.14±13.53	31.71±12.47
	Aerobic exercise	38.29±10.65	38.29±12.20
	Resistance training	32.24±10.49	32.24±10.12
LDL-C (mg/dL)	Control	60±12.57	60.14±12.77
	Aerobic exercise	48.57±60	48.14±9.51
	Resistance training	51.29±27.95	51.14±26.32
Total cholesterol (mg/dL)	Control	153.29±31.61	131.57±31.41
	Aerobic exercise	131.14±33.58	130.57±34.21
	Resistance training	127.29±22.81	126.86±22.62

Values are Mean ±SD; spKt/V: single pool kt/V; hs-CRP: highly sensitive C-reactive protein; HDL-C: high density lipoprotein cholesterol; LDL-C: low density lipoprotein cholesterol

## Discussion

Resistance training is a form of strength training in which each effort is performed against a specific opposing force generated by resistance (i.e., resistance to being pushed, squeezed, stretched, or bent). Exercises are isotonic if a body part is moving against the force. Exercises are isometric if a body part is holding still against the force. Resistance exercise is used to develop the strength and size of skeletal muscles. Properly performed, resistance training can provide significant functional benefits and improvement in overall health and well-being. Resistance training should not be confused with weightlifting, power lifting, or body buildings, which involve different types of strength training with nonelastic forces such as gravity rather an immovable resistance. Full range of motion is important in resistance training because muscle overload occurs only at the specific joint angles where the muscle is worked.[18]

We considered this issue in our study and designed the resistance training in three sets with regard to full range of motion. The study of fat metabolism and acute resistance exercise in trained men, conducted by East Carolina University, found that resistance exercise is more beneficial than aerobic exercise for fat loss.[19]

Aerobic training (e.g., stationary cycling) is an exercise that involves or improves oxygen consumption in the body metabolic or energy generating processes.[20] Aerobic exercise is more beneficial in atherosclerosis reduction, insulin sensitivity improvement, and raising HDL–C.[21–23] Resistance exercise improves insulin resistance, muscular strength and endurance, enhances flexibility, alters body composition (particularly increases fat loss), and also decreases risk factors for cardiovascular disease.[19,24,25] In addition, aerobic exercise and resistance training have been reported to have a beneficial influence on functional capacity, quality of life, cardiovascular risks factors, anemia, lipid levels, insulin resistance, and inflammatory cytokines in ESRD patients.[12,21]

Intradialytic aerobic exercise has been shown to be safe in the first 2 h of dialysis; after 2 h, cardiac decompensation may occur.[26] Furthermore, intradialytic cycling improves hematocrit levels, peak oxygen consumption, quality of life, dialysis efficacy, and physical functioning,[15,27] while interdialytic aerobic training on nondialysis days improves quality of life, lipid profile, anemia, and insulin sensitivity,[28,29] and decreases anxiety-depression disorders.[30]

It has been reported that intradialytic resistance training increases muscular strength without increase in lean body mass[31] and improves physical functioning, while interdialytic resistance exercise increases functional performance, quality of life, and strength.[32]

Although, interdialytic exercise has been suggested to be superior to intradialytic exercise due to better effect on aerobic capacity,[33] patients generally have greater difficulty with interdialytic prescription and intradialytic exercise has fewer dropouts.[14,33] For this reason and in order to improve patients’ compliance to maintain on training program, provide motivation in a structured environment, and facilitate patients’ monitoring, we preferred to design intradialytic exercise training, while the majority of studies have focused on interdialytic prescription.

Compared with other studies, we found that aerobic and resistance exercises within 8 weeks had favorable effects on chronic inflammatory status in HD patients, so that aerobic exercise induced more hs-CRP level reduction (83.42% vs. 67.89%) than resistance training.[12,21,32,34–36] This effect may be ascribed to longer duration and more continuity of aerobic exercise in the current study or nature of this type of exercise.

Some studies have reported that exercise has improved solute removal due to increased muscle blood flow and open capillary surface area associated with increased flux of urea from the tissue to the vascular compartment during exercise and increased cell membrane permeability to water-soluble molecules such as creatinine due to rising exercise-induced body temperature.[7,15,37] However, these findings were limited only to aerobic exercises which took at least 1 h. Serum creatinine reduction in our study must be regarded cautiously because dialysate creatinine concentration and residual renal function during exercise programs were not measured (before starting exercise programs, the participants had no urine output). On the other hand, serum urea and *Kt/V* had no significant changes and indicated that solute removal did not change in this study. We have no convincing explanation for this finding except occurrence of mild rhabdomyolysis due to exertion which resulted in serum creatinine increment, in comparison to urea. Thus, increased muscle blood flow and open capillary surface area comparatively reduced serum creatinine level more than urea. It is notable that solute removal during HD focuses on urea and not on creatinine.

As mentioned previously, these two exercise programs had no beneficial effects on lipid profiles. Although, one investigation has shown that interdialytic aerobic exercise has been associated with triglyceride decrease and HDL elevation,[29] however there is lack of sufficient evidences regarding the efficacy of intradialytic prescription and resistance training in serum lipid reduction.[38–40] To our knowledge, interdialytic aerobic exercise results in lipid profile improvement due to weight loss, an effect which has not been reported with intradialytic exercise up to now probably because of short duration and interrupted nature of this exercise. As mentioned above, in this study no significant changes in body mass indices were seen among different groups during 8 weeks, which was attributed to short duration of the study. Furthermore, there were multiple factors such as gender, nutritional status, uncontrolled secondary hyperparathyroidism which inhibit lipolytic activity, dialysis membrane bioincompatibility resulting in lipid peroxidation and drug consumption that could contribute to exercise ineffectiveness. Because of short duration of our study, its intradialytic design and merely men’s participation the latter findings about serum lipid levels alterations during exercise (particularly aerobic) must be regarded cautiously.

As mentioned above, we encountered many limitations in current study including refusal of female patients for participation because of religious beliefs and lack of enough place for gender separation during exercise, small sample size due to lack of motivation, noncompliance of patients for additional interdialytic exercise program design and longer duration exercise training, with respect to low motivation and few prior studies that have been conducted regarding exercise training in HD units throughout Iran and limitation for creatinine measurement in dialysate fluid.

Furthermore, there is a lack of information about benefits of exercise during HD and intervals between each dialysis session which prevents HD patients from exercising more regularly.

## Conclusion

This study showed the effects of two different quantitative exercise interventions that could be safely administered in the HD units to a wide range of the dialysis population. Despite the beneficial effects of aerobic exercise training in the HD population, there is a lack of the widespread adoption of such program. This may attributed to nephrologists’ distrust to the reported results because of small sample sizes or uncontrolled trials and conflict with the generalizability of these benefits due to vigorous nature, low practicality of the training programs, and the highly selected patients.

We recommend that nephrologists must consider and implement such simple training programs as standard clinical practice in HD units and expand these programs to nondialysis days particularly in compliant patients throughout the world. Furthermore, additional studies are needed with longer duration, larger sample sizes, and different gender regarding exercise (particularly combined aerobic and resistance trainings) in HD patients.
